# IDH mutant and 1p/19q co-deleted oligodendrogliomas: tumor grade stratification using diffusion-, susceptibility-, and perfusion-weighted MRI

**DOI:** 10.1007/s00234-017-1839-6

**Published:** 2017-05-04

**Authors:** Yu Lin, Zhen Xing, Dejun She, Xiefeng Yang, Yingyan Zheng, Zebin Xiao, Xingfu Wang, Dairong Cao

**Affiliations:** 1 Department of Radiology, First Affiliated Hospital of Fujian Medical University, 20 Cha-Zhong Road, Fuzhou, Fujian 350005 People’s Republic of China; 2 Department of Pathology, First Affiliated Hospital of Fujian Medical University, Fuzhou, China

**Keywords:** IDH mutation, 1p/19q co-deletion, Oligodendrogliomas, SWI, DSC-PWI

## Abstract

**Purpose:**

Currently, isocitrate dehydrogenase (IDH) mutation and 1p/19q co-deletion are proven diagnostic biomarkers for both grade II and III oligodendrogliomas (ODs). Non-invasive diffusion-weighted imaging (DWI), susceptibility-weighted imaging (SWI), and dynamic susceptibility contrast perfusion-weighted imaging (DSC-PWI) are widely used to provide physiological information (cellularity, hemorrhage, calcifications, and angiogenesis) of neoplastic histology and tumor grade. However, it is unclear whether DWI, SWI, and DSC-PWI are able to stratify grades of IDH-mutant and 1p/19q co-deleted ODs.

**Methods:**

We retrospectively reviewed the conventional MRI (cMRI), DWI, SWI, and DSC-PWI obtained on 33 patients with IDH-mutated and 1p/19q co-deleted ODs. Features of cMRI, normalized ADC (nADC), intratumoral susceptibility signals (ITSSs), normalized maxim CBV (nCBV), and normalized maximum CBF (nCBF) were compared between low-grade ODs (LGOs) and high-grade ODs (HGOs). Receiver operating characteristic curve and logistic regression were applied to determine diagnostic performances.

**Results:**

HGOs tended to present with prominent edema and enhancement. nADC, ITSSs, nCBV, and nCBF were significantly different between groups (all *P* < 0.05). The combination of SWI and DSC-PWI for grading resulted in sensitivity and specificity of 100.00 and 93.33%, respectively.

**Conclusions:**

IDH-mutant and 1p/19q co-deleted ODs can be stratified by grades using cMRI and advanced magnetic resonance imaging techniques including DWI, SWI, and DSC-PWI. Combined ITSSs with nCBV appear to be a promising option for grading molecularly defined ODs in clinical practice.

## Introduction

Oligodendrogliomas (ODs) are glial neoplasms originated from oligodendrocytes that primarily affect supratentorial parenchyma [1]. These entities were previously classified into grades II and III according to the 2007 World Health Organization (WHO) classification system of the central nervous system (CNS). The traditional and indispensable approach relies on the histopathologic analysis of proliferative and mitotic activity, nuclear atypia, and cellularity as significant predictors of disease progression [2, 3].

Historically, co-deletion of whole chromosome arms 1p and 19q, namely, 1p/19q co-deletion, has proved to be a diagnostic and prognostic biomarker of ODs [4–6]. However, progression-free survival (PFS) and overall survival (OS) of ODs between grades are dramatically different after adjustment for genotypes (defined by 1p/19q co-deletion) [7]. Isocitrate dehydrogenase (IDH) mutation is frequently found in WHO grade II–III oligodendrogliomas and astrocytomas [8, 9], and is currently implicated as a prerequisite of tumorigenesis for some types of diffuse gliomas and a precondition of 1p/19q co-deletion [10, 11]. According to the 2016 WHO classification system, the “integrated diagnosis” of ODs requires histological classification, WHO grade, and molecular information (both IDH mutation and 1p/19q co-deletion) [12, 13]. An oligodendroglioma-like tumor lacking diagnostic mutations is given a “not otherwise specified (NOS)” designation, which is strongly discouraged by neuro-oncologists. When dealing with NOS ODs, care should be taken throughout to exclude CNS tumors including astrocytomas, glioblastomas, clear cell ependymoma, and dysembryoplastic neuroepithelial tumor [12]. In addition, nearly all histological “oligoastrocytomas” can be reclassified as either astrocytomas or ODs using genetic testing [12, 14].

Diffusion-weighted imaging (DWI) could reflect the Brownian movement of water molecules and the cytogenetic profile of cerebral tumors through the measurement of apparent diffusion coefficient (ADC) values [15, 16]. As another advanced magnetic resonance imaging (aMRI) approach, susceptibility-weighted imaging (SWI) utilizes intratumoral susceptibility signals (ITSSs) to demonstrate tumor vascularity and blood metabolite sensitively [17, 18]. Regional cerebral blood volume (rCBV) and regional cerebral blood flow (rCBF) derived from dynamic susceptibility contrast perfusion-weighted imaging (DSC-PWI) provide hemodynamic information of microvasculature in glioma grading and genotyping [19–24]. Non-invasive measurement of rCBV has been confirmed to correlate with tumor vascularity and clinical prognosis of gliomas, which has the potential to provide additional information to conventional MRI (cMRI) [7, 19, 21]. As recently revealed, IDH mutations or 1p/19q co-deletions correlate with aberrant cellularity and angiogenesis by regulating proliferation and vascularization [6, 19, 21, 25]. Therefore, we suspected that the inclusion of mixed genetic profiles in previous studies might lead to a low prediction accuracy of aMRI techniques for grading ODs [7, 26].

During the past two decades, extensive studies have focused on the surrogate MRI characters of tumor grade (defined by histopathology) and genetic subtype (defined by 1p/19q co-deletion or IDH mutation) of CNS tumors [19, 23, 24, 27, 28], whereas these two genic alterations act as diagnostic markers rather than grouping factors for ODs [12]. Thus, it is inappropriate for the neuropathologists or neuroradiologists to “genotype” ODs following the WHO update. To our knowledge, there is no aMRI research in presurgical grading of the redefined tumors. Therefore, the present study aims to investigate whether multiparametric MRI is predictive of tumor grades of the new entity.

## Methods

### Patients

This retrospective study was approved by the institutional review board, and patient informed consent requirement was waived. Patients with oligodendroglial tumors were histologically confirmed through image-guided stereotactic biopsy or tumor biopsy on resection performed at our hospital from January 2014 to January 2017. The histopathologic diagnosis was made by experienced neuropathologists according to the updated WHO classification standards. The inclusion criteria were (1) a histopathology diagnosis of ODs or oligoastrocytomas according to 2007 WHO classification; (2) 3.0 Tesla cMRI scans combined with DWI, SWI, and DSC-PWI before any intervention; and (3) a known IDH mutation and 1p/19q co-deletion status for reclassification according to 2016 WHO guidelines.

### Conventional MRI

The MR examinations were performed in the routine clinical workup using a 16-channel dedicated head matrix coil on a 3.0 Tesla MRI system (MAGNETOM Verio; Siemens Healthcare, Erlangen, Germany). The sequences of cMRI protocols were gradient-echo T1-weighted imaging (T1WI, TR/TE = 250 ms/2.48 ms), turbo spin-echo T2-weighted imaging (T2WI, TR/TE = 4000 ms/96 ms), fluid-attenuated inversion recovery (FLAIR, TR/TE = 9000 ms/94 ms, TI 2500 ms) imaging, and gradient-echo contrast-enhanced T1WI (CE-T1WI, TR/TE = 250 ms/2.48 ms). All images were acquired with field of view (FOV) of 220 mm × 220 mm, and slice thickness of 5 mm.

### Advanced MRI

DWI was performed in the axial plane with a spin-echo-planar sequence (TR/TE = 8200/102 ms, NEX = 2.0). Corresponding ADC maps were generated automatically by the MRI system. Diffusion gradients were encoded in three orthogonal directions to accentuate the effects of diffusion.

Non-enhanced SWI was performed using a 3D fully flow-compensated gradient-echo sequence (TR/TE = 27/20 ms, flip angle = 15°) and a reconstruction by combining the magnitude and phase images.

DSC-PWI was achieved with a gradient-recalled echo-planar sequence (TR/TE = 1000–1250/54 ms, flip angle = 35°, NEX = 1.0). In the first three phases, non-enhanced images were scanned to establish a precontrast baseline. For the scan in the fourth phase of DSC-PWI, a standard dose (0.1 mmol/kg) of gadobenate dimeglumine (Gd-BOPTA) was injected intravenously at a flow rate of 3.00 ml/s through a catheter inserted into the antecubital vein, followed by a 20-ml continuous saline flush. A series of 60 phases and a total of 1200 images were constantly produced in 96 s.

### Image analysis

All imaging assessments were performed with the standard software integrated in the MRI system including syngo.MR Neuro Perfusion Engine (syngo.via; Siemens Healthcare, Erlangen, Germany). Two neuroradiologists (with 30 and 9 years of experience, respectively) were blinded to the pathological features, and the cMRI sequences were evaluated individually. The senior made the final decision when there was disagreement between them. The following characteristics were qualitatively evaluated: tumor location (assessed by primary lobe of involvement; frontal, parietal, temporal, occipital, insular, and other sites), signal intensity (homogeneous vs. heterogeneous), tumor borders (sharp/smooth vs. indistinct/irregular); peritumoral edema (presence vs. absence), and contrast enhancement pattern (no/blurry vs. nodular/ring-like).

To assess DWI data, ADC values were measured by manually placing regions of interest (ROIs) on the ADC maps. At least five non-overlapping ROIs (size 25–40 mm^2^) were placed inside the tumor areas of visually lowest ADC. The minimum ADC (ADC_min_) was calculated as the mean value from the ROI of the lowest ADC. The ROIs’ placement was made from the solid portion (defined on FLAIR images and CE-T1WI) to avoid hemorrhagic, calcified, necrotic, and cystic regions that might affect the measurements. To minimize variances in ADC_min_ values, normalized ADC (nADC) was derived from the ratios of the tumor ADC_min_ to the mean ADC of the contralateral normal-appearing white matter (CNWM, defined on T2WI and CE-T1WI).

Minimum intensity projection technique at 2-mm slice thickness was conducted for the semi-quantitative analysis of SWI. Intratumor calcification was identified and excluded using phase image cross-correlation analysis or corresponding CT scans. The degree of ITSSs included four grades as previously described [29, 30]: (1) grade 0 with no ITSS; (2) grade 1 with 1–5 dot-like or linear ITSSs; (3) grade 2 with 6–10 dot-like or linear ITSSs; and (4) grade 3 with more than 11 dot-like or linear ITSSs.

To evaluate DSC-PWI data, rCBV and rCBF maps were generated automatically using a single-compartment model and an arterial input function. Each value was measured using the same method in the previous nADC assessments by targeting high rCBV/rCBF. At least five non-overlapping ROIs were placed inside the tumor areas of visually highest rCBV/rCBF. The maximum CBV/CBF was calculated as the mean value from the ROI of the greatest rCBV/rCBF. The normalized maximum rCBV (nCBV) and normalized maximum rCBF (nCBF) were calculated by normalizing to the CNWM.

All the parameters derived from DWI and DSC-PWI were measured by a qualified neuroradiologist who was experienced in image processing and analysis, and blinded to the tumor pathology. The methods used in the analysis of DWI, SWI, and DSC-PWI have been proven to provide the highest interobserver and intraobserver reproducibility [27, 29, 30].

### Molecular studies

Immunohistochemistry (IHC) staining was performed using 5-μm-thick sections in paraffin-embedded specimens and anti-IDH1 (R132) (clone DIA-H09, Dianova Inc., Hamburg, Germany) at a dilution of 1:500. Samples were scored positive when IDH1-R132 staining showed more than 10% of the tumor cells.

IDH1/2 alterations in the hotspot codons R132 and R172 were also simultaneously assessed using bidirectional cycle sequencing of polymerase chain reaction (PCR)-amplified fragments with the standard Sanger method.

1p/19q deletions were detected by fluorescence in situ hybridization (FISH) analysis on 5-μm-thick paraffin-embedded sections using Vysis 1p36/1q25 and 19q13/19p13 dual-color probes according to the manufacturer’s instructions (Vysis Inc., Illinois, USA). Signals were scored with at least 100 non-overlapping intact nuclei. 1p/19q co-deletion was defined as the signal ratio of 1p/1q <0.70 and 19q/19p <0.70.

### Statistical analyses

Quantitative data were described as mean ± standard deviation (SD). Interobserver variabilities of cMRI features and ITSSs levels were analyzed by kappa statistics. Parameters derived from aMRI between low-grade ODs (LGOs) and high-grade ODs (HGOs) were compared using Mann-Whitney *U* test. Either Pearson’s chi-square test or Fisher’s exact test was performed for categorical data. The receiver operating characteristic (ROC) curves and binary logistic regression analysis were carried out to decide diagnostic accuracy and optimum cutoff value. The sensitivity, specificity, positive predictive value (PPV), negative predictive value (NPV), Youden index (YI), and area under the curve (AUC) were calculated based on the optimal threshold for each parameter and combination. Statistical analysis was conducted using Statistical Package for the Social Sciences (SPSS), version 19.0.0 (SPSS Inc., Chicago, IL, USA), and MedCalc, version 12.1.0 (MedCalc Inc., Mariakerke, Belgium). A significant difference was defined as a *P* value less than 0.05.

## Results

Thirty-three cases (17 males and 16 females, age 42.97 ± 9.03 years) were diagnosed as IDH-mutant and 1p/19q co-deleted ODs based on the current WHO criteria. DWI, SWI, and DSC-PWI combined with cMRI were performed on all patients. Five cases of DSC-PWI were of poor perfusion quality and excluded in further analysis.

Table 1 shows the summarization of the main clinical and cMRI features of molecularly defined (IDH-mutant and 1p/19q co-deleted) ODs between groups. All kappa coefficients of interobserver measurements were greater than 0.75 (an excellent agreement). Out of 33 patients, 11/18 (61.1%) LGOs and 13/15 (86.7%) HGOs situated in the frontal lobe with insignificant difference between grades. Heterogeneous appearance (including hemorrhage and cystic degeneration) and infiltrating borders were non-specifically observed in ODs regardless of grades. It is noteworthy that LGOs revealed a decreased level of edema compared to anaplastic counterparts (*P* = 0.005). Additionally, HGOs were related to the nodular or ring-like enhancement patterns on CE-T1WI (*P* = 0.002).

**Table 1:** The main clinical and cMRI features of LGOs and HGOs

	LGO (*n* = 18)	HGO (*n* = 15)	Total	*P* value
Gender (male/female)	9/9	8/7	17/16	0.849
Age (years)	41.56 ± 9.03	44.67 ± 9.05	42.97 ± 9.03	0.319
Location				0.134
Frontal	11	13	24	
Parietal	2	1	3	
Temporal	2	0	2	
Occipital	1	0	1	
Insular	2	0	2	
Others	0	1	1	
Signal				0.134
Homogeneous	7	2	9	
Heterogeneous	11	13	24	
Border				0.722
Sharp/smooth	6	4	10	
Indistinct/irregular	12	11	23	
Edema				*0.013*
Presence	6	12	18	
Absence	12	3	15	
Enhancement				*0.005*
No/blurry	14	4	18	
Nodular/ring-like	4	11	15	

A italicized *p* value indicate a significant difference between results (*p* < 0.05)

**Fig. 1:**
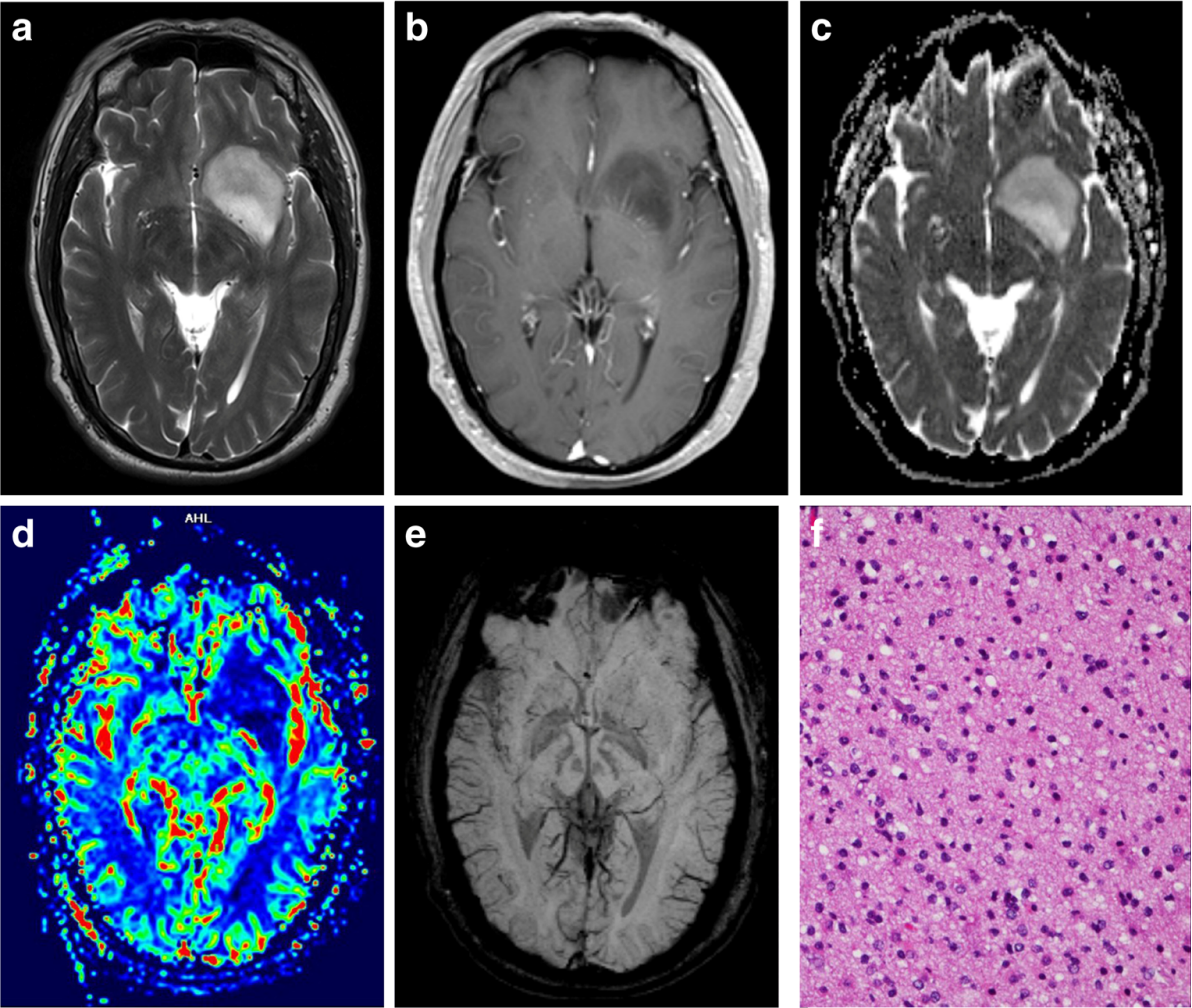
A 55-year-old man with molecularly defined LGO. On axial T2WI (**a**), the lesion shows homogeneous signal. Axial CE-T1WI (**b**) demonstrates absence of contrast enhancement. Corresponding ADC maps (**c**) show an increased ADC value (nADC = 1.52). rCBV maps (**d**) show significantly reduced perfusion with the calculated nCBV of 1.12. SWI (**e**) presents nearly no evidence of ITSS. H&E staining (×100) photomicrograph (**f**) reveals a low cell density

**Fig. 2:**
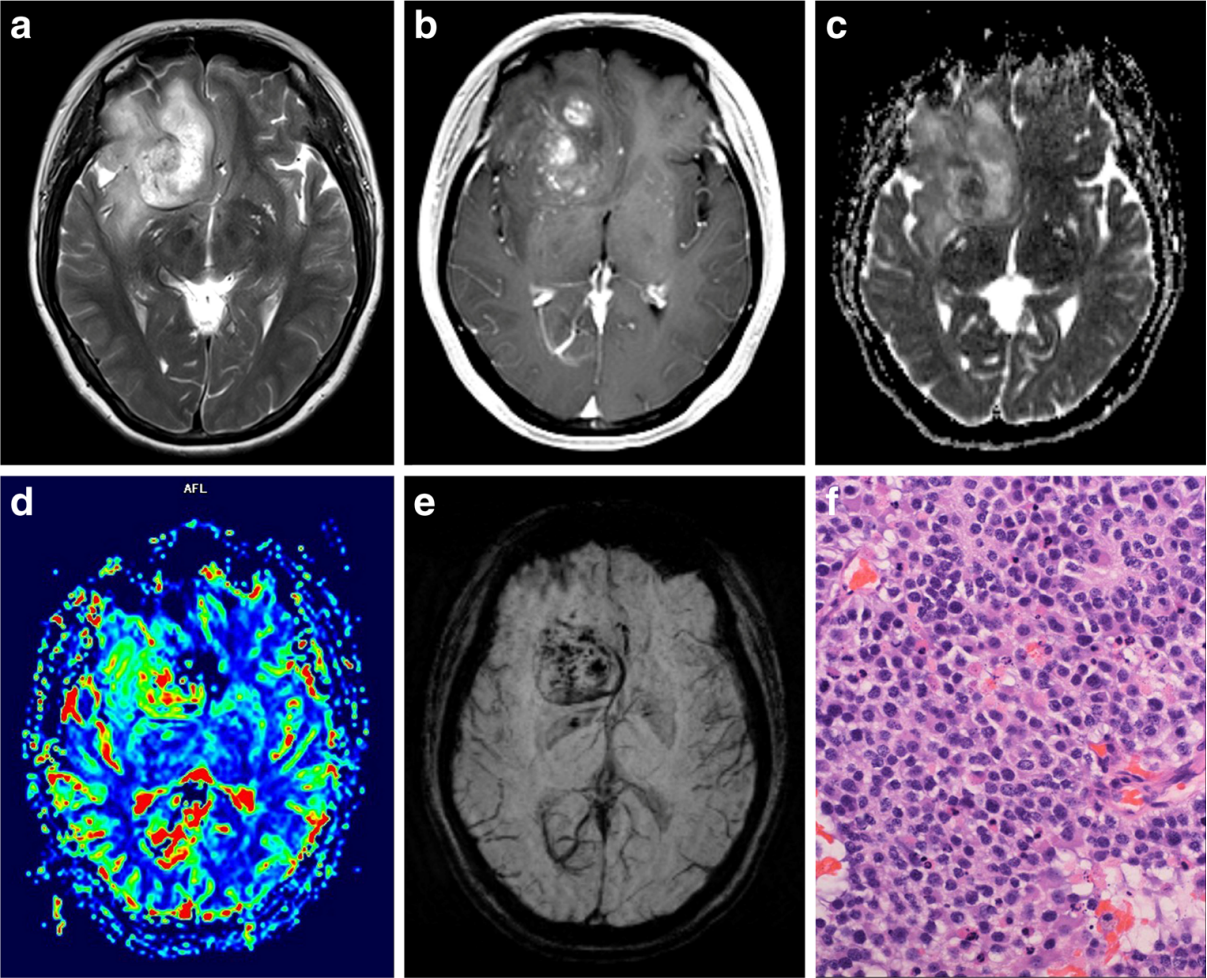
A 50-year-old woman with molecularly defined HGO. On axial T2WI (**a**), the lesion shows heterogeneous signals with possible cystic or necrotic regions. Markedly nodular contrast enhancement is demonstrated on CE-T1WI (**b**). Corresponding ADC maps (**c**) exhibit a partially decreased ADC value (nADC = 0.85). rCBV maps (**d**) show significantly elevated perfusion with a nCBV value of 4.69. SWI reveals (**e**) a maximum degree of ITSSs. H&E staining (×400) photomicrograph (**f**) reveals relatively high cellularity and proliferative vessels

Tables 2 and 3 give the (semi-)quantitative measurements of aMRI for LGOs and HGOs. Relevant MRI images are depicted in Figs. 1 and 2. nADC values from DWI were significantly higher in LGOs than in HGOs (*P* < 0.001). ITSS levels of HGOs were substantially greater than that of LGOs with seven (46.7%) HGOs which exerted an agglomerated or nodular appearance of ITSSs (*P* = 0.006). Both nCBV and nCBF values in HGOs were significantly higher than the values of LGOs (*P* < 0.001 and *P* = 0.005).

**Table 2:** Comparison of variables derived from DWI and DSC-PWI

	LGO (mean ± SD)	HGO (mean ± SD)	***P*** value
nADC	1.36 ± 0.27	0.96 ± 0.12	*<0.001*
nCBV	2.34 ± 1.16	5.08 ± 1.81	*<0.001*
nCBF	2.81 ± 1.26	5.45 ± 2.64	*0.005*

A italicized *p* value indicate a significant difference between results (*p* < 0.05)

**Table 3:** Comparison of ITSS levels derived from SWI (*P* = 0.006)

ITSS level	LGOs	HGOs	Total
0	7	1	8
1	8	5	13
2	1	3	4
3	2	6	8
Total	18	15	33

The results of ROC analysis of the parameters are illustrated in Table 4. Optimal cutoff values of quantitative nADC from DWI and quantitative nCBV from DSC-PWI in tumor grading were determined as 1.11 and 2.90, respectively. The sensitivity/specificity of nADC and nCBV were 100.00/77.78 and 92.31/80.00%, respectively. With a threshold value of ≥1 for semi-quantitative ITSSs from SWI, the sensitivity and specificity in the diagnosis of HGOs were 60.00 and 83.33%, respectively.

**Table 4:** Cutoff, sensitivity (%), specificity (%), PPV (%), NPV (%), YI, and AUC of parameters for grading OD

	Cutoff	Sensitivity	Specificity	PPV	NPV	YI	AUC
cMRI		73.33	77.78	72.97	78.09	0.51	0.76
nADC (DWI)	1.11	100.00	77.78	78.64	100.00	0.78	0.92
ITSS (SWI)	1	60.00	83.33	74.65	71.80	0.43	0.77
nCBV (DSC-PWI)	2.90	92.31	80.00	79.06	92.71	0.72	0.90
DWI + SWI		73.33	100.00	100.00	82.09	0.73	0.95
DWI + DSC-PWI		92.31	93.33	91.89	93.68	0.86	0.94
SWI + DSC-PWI		100.00	93.33	92.46	100.00	0.93	0.99
aMRI		100.00	93.33	92.46	100.00	0.93	0.99

*PPV* positive predictive value, *NPV* negative predictive value, *YI* Youden index, *AUC* area under the curve

Logistic regression and ROC analyses were conducted for cMRI (combining edema and enhancement) and aMRI (combining DWI, SWI, and DSC-PWI by two or three parameters). The combination of SWI and DSC-PWI resulted in the highest sensitivity and specificity of 100.00 and 93.33%, respectively. Nevertheless, combined DWI, SWI, and DSC-PWI techniques yielded similar diagnostic performance.

## Discussion

Significant concordance between the histopathological grading and aMRI-based grading was found in our reclassified cases. This concordance may be of more clinical value as we followed the updated WHO classification standards which have already redefined many infiltrating gliomas. Our study has revealed non-invasive MRI techniques including DWI, SWI, and DSC-PWI which may improve the diagnostic accuracy in grading redefined ODs.

Although molecular and genetic factors have been introduced recently, a large body of evidence has suggested that glioma grading based on histopathological evaluation is still important for determining therapeutic options and predicting clinical outcomes [2, 5, 12, 31, 32]. Shaw et al. suggest that OS (approximately 4 years of HGOs and 10 years of LGOs) and treatment planning are different between grades [31–33]. Patients with LGOs can benefit from subtotal resection and early radiotherapy with small fraction doses, or remain stable without any treatment [5, 34, 35]. In contrast, several randomized controlled trials of HGOs show that adjuvant chemotherapy increases PFS while postoperative radiotherapy produces minimum benefit [33, 36]. Besides, preoperative MRI-based grading and guidance may affect the surgical strategy to maximize safe resection [31, 37].

Frontal location, characteristic calcification, poorly defined border, and internal heterogeneity are highly suggestive of molecularly defined ODs as unveiled in the latest studies [27, 38–41], which may serve as reliable and robust biomarkers in the differential diagnosis prior to tumor grading. The results of this study display that molecularly defined ODs have a predilection for the frontal lobes that is consistent with previous findings [27]. Peritumoral edemas in HGO patients are frequently encountered in our study, mainly attributed to the high frequency of cellular/vasogenic pathogenesis in high-grade tumors [42]. Up to date, Johnson et al. have suggested that the tumor borders of molecularly defined ODs are fairly infiltrative [41, 43]. While peritumoral edema was determined based on its non-enhancement on CE-T1WI and higher signal on FLAIR in contrast to clearly enhanced tumor solid component, radiologically, definite differentiation of tumor infiltration from peritumoral edema is still a technical challenge in the field. Although nodular or ring-like enhancement in association with blood-brain barrier breakdown and angiogenesis [37, 42] is frequently observed in HGOs, it should be further noted that such enhancement patterns of HGOs are more homogenous than that of glioblastoma (WHO grade IV, most are IDH wild types), whereas it is quite difficult to stratify tumor grades by utilizing cMRI only.

Although nADC values were not significantly different between WHO grades in morphologically defining ODs (based on WHO 2007 criteria) [26], molecularly defined ODs could be stratified by grades applying nADC as shown in our research. Diffusion MRI enables studies in tumorous physical properties microscopically: increased proliferation elevates cellularity and restricts water diffusion [15, 44]. Mutations of the IDH gene family produces oncometabolite 2-hydroxyglutarate, leading to a slower growth of tumor cells compared to the wild types [9]. Conversely, a poor correlation between 1p/19q co-deletion and cell proliferation was confirmed in an early study [27]. Consequently, it is reasonable to speculate that so-called IDH wild-type ODs of various proportions in both grades may play as confounding factors and result in a complexity of heterogeneity in preceding studies.

SWI appears to be a promising imaging method in grading gliomas based on the different frequencies and appearances of ITSSs [18]. The dot-like hypointense signal is regarded as microhemorrhage and the linear signal as an intralesional vessel, both of which are common in malignant CNS tumors [30]. Similarly, the results of our study indicate a significant difference in ITSSs between grades, suggesting that ITSSs can be a valuable biomarker for a precise diagnosis of HGO. However, medium-to-high levels of ITSSs in LGOs were occasionally observed, which may be attributed to the moderately increased neovascularity in both LGOs and HGOs as a consequence of 1p/19q co-deletion [21, 40].

Our study introduced multiple diagnostic parameters derived from DSC-PWI to identify high-grade IDH-mutant and 1p/19q co-deleted ODs. We believe that nCBV and nCBF values can be used as surrogates of angiogenic features of molecularly defined ODs. A significant positive correlation was found between tumor grades and nCBV values. However, most of the previous researchers [7, 24, 25, 28, 45] indicated that nCBV was either inefficient or overlapping in grading ODs when using the gold standard of 2007 WHO criteria. With improved understanding of the pathogenic mechanism, scientists suggest that IDH mutation can reduce the activation of hypoxia-inducible factor-1 alpha and inhibit downstream signaling pathways of angiogenesis [19, 46]. Similarly, some studies indicated that gliomas with 1p/19q co-deletion were likely to have mildly elevated rCBV due to increased microvascular proliferation [21, 25]. Indeterminate proportions of “genotypes” may substantially increase the overlap of perfusion parameters between LGOs and HGOs suggested in previous studies [7, 25].

The combination of ITSSs and nCBV remarkably improved the grading accuracy, and may serve as an optimal method neuroradiologically. However, the combination of all aMRI methods provided no improvement in differentiating anaplastic tumors from LGOs. Although nADC is an ideal parameter for grading molecularly defined ODs, additional DWI procedures may not be necessary when both SWI and DSC-PWI are available.

There are some limitations in our study such as the retrospective nature and small sample. Although the case number of IDH-mutant and 1p/19q co-deleted OD is relatively limited due to its low prevalence and the complexity of multiple cytogenetic characteristics required for diagnosis, further study is needed in multicenter large-scale MRI-based radiomics approaches. Moreover, a possible contrast extravasation in DSC-PWI may generate an imprecise estimation of glioma nCBV. Both preload of gadolinium and leakage correction may be necessary to minimize the leakage contamination. In addition, intratumoral calcific, cystic, necrotic, and hemorrhagic areas may potentially interfere with our manual measurements despite the efforts made to minimize the interference. Finally, a long-term follow-up is desired for analysis of patients’ prognosis.

## Conclusions

The study demonstrates that IDH-mutant and 1p/19q co-deleted ODs can be stratified by grades using cMRI and aMRI techniques including DWI, SWI, and DSC-PWI with high specificity and sensitivity. HGOs are prone to prominent edema and enhancement. Furthermore, adding ITSSs to nCBV in clinical practice seems to be a promising option for grading molecularly defined ODs.
